# Isolation and chemical characterization of the biosurfactant produced by *Gordonia* sp. IITR100

**DOI:** 10.1371/journal.pone.0264202

**Published:** 2022-04-14

**Authors:** Arif Nissar Zargar, Sarthak Mishra, Manoj Kumar, Preeti Srivastava

**Affiliations:** 1 Department of Biochemical Engineering and Biotechnology, Indian Institute of Technology Delhi, New Delhi, India; 2 Indian Oil Corporation, R&D Centre, Faridabad, India; Universidad Autonoma de Chihuahua, MEXICO

## Abstract

Biosurfactants are amphipathic molecules produced from microorganisms. There are relatively few species known where the detailed chemical characterization of biosurfactant has been reported. Here, we report isolation and chemical characterization of the biosurfactant produced by a biodesulfurizing bacterium *Gordonia* sp. IITR100. Biosurfactant production was determined by performing oil spreading, drop-collapse, Emulsion index (E_24_), and Bacterial adhesion to hydrocarbons (BATH) assay. The biosurfactant was identified as a glycolipid by LCMS and GCMS analysis. The chemical structure was further confirmed by performing FTIR and NMR of the extracted biosurfactant. The emulsion formed by the biosurfactant was found to be stable between temperatures of 4°C to 30°C, pH of 6 to 10 and salt concentrations up to 2%. It was successful in reducing the surface tension of the aqueous media from 61.06 mN/m to 36.82 mN/m. The biosurfactant produced can be used in petroleum, detergents, soaps, the food and beverage industry and the healthcare industry.

## Introduction

Biosurfactants are a biologically-produced group of structurally diverse amphipathic molecules capable of assembling at the interface between two phases with degrees of polarity differing from each other [1]. These interfaces may be liquid/liquid (oil-water), liquid/gas (water-air) or solid/liquid (metal-water) [2]. The amphipathic nature of biosurfactants makes them proficient in forming microemulsions by reducing the interfacial tension between two phases and therefore enhances solubility of one phase in another [1,3–5]. A typical biosurfactant contains a hydrophilic part composed of anions/cations, hydrophilic amino acids or a sugar unit, while the hydrophobic part is composed of hydroxylated, saturated or unsaturated fatty acids (lipids) or hydrophobic peptides [3]. Most commonly reported biosurfactants are glycolipids in which long-chain fatty acids are coupled with carbohydrates. Other biosurfactants which include lipopeptides, heteropolysaccharides and lipopolysaccharides are relatively more complex in structure [1]. Biosurfactants have fostered interest in the past three decades due to significant advantages that they offer over their chemical counterparts. These include: low toxicity, high biodegradability, higher foaming, environmental compatibility, ability to form micelles at low Critical micellar concentration, low cost of production, and higher specific activity and selectivity across a wide range of conditions including extremes of pH, temperature and salt concentration [1,3,6–9]. Biosurfactants also have antibacterial, antiviral and antifungal properties [1,10–12]. These properties make them versatile in terms of their applications and therefore are used in several industries such as agriculture [10], food [6,13,14], petroleum [15], cosmetic [16], healthcare [17–19], soaps and detergent [19], nanotechnology [8,20,21], paper and pulp [1,22], coal [17,23] and ceramics [1,24]. Major applications include bioremediation (oil spills, groundwater contaminated with hydrocarbons, sites with heavy metals and other pollutants) [17,25–29], cleaning oil sludge in tanks, microbial enhanced oil recovery (MEOR) [2,13,30] and replacement of chlorinated cleaning solvents [31].

In 2017, the global biosurfactant market was evaluated at over USD 1.85 billion with consumption of over 540 kilo tons predicted by 2024 [32]. Despite large demand alongside numerous advantages, their utilization is limited by the relatively higher cost of production and 10 to 40-fold lower CMC compared to chemical counterparts. Various strategies for biosurfactant production such as solid-state fermentation, biosurfactant co-production, specific yield augmentation by media modulations and immobilization for growth enhancement have been suggested for decreasing production costs [19]. Yet choice of the microorganism is a key factor in reducing the cost. A variety of biosurfactant-producing microorganisms have been listed by Banat [3] but the number of species remains low and those characterised need improvement in terms of production efficiency and versatility of growth substrate. There is therefore a requirement for biosurfactant production in new microbial species.

Here, we report the identification and characterization of the biosurfactant produced by *Gordonia* sp. IITR100. The strain was previously isolated in our lab from petroleum-contaminated soil by enrichment culture technique using 4,6-dimethyl dibenzothiophene as the sole sulphur source [33]. The bacterium has previously been characterized for its heavy oil biodesulfurization and viscosity reduction potential [34]. It has been reported to desulfurize 76% sulfur from crude oil and also reduces the viscosity of heavy crude oil by 31%. Both the processes are restrained by the limited availability of the polyaromatic hydrocarbons to the degrading microbes. Identification and Characterization of the biosurfactant will help in designing strategies for minimizing the mass transfer limitations and designing the process such that the microbes will have a maximum access to the hydrocarbon fraction. This will help in enhancing and improving heavy oil biodesulfurization and viscosity reduction. Most species of the genus *Gordonia* have the capability to degrade environmental pollutants, xenobiotics and biodegradable natural polymers [35]. Multiple experiments including drop-collapse, microplate assay, stable emulsion index (E_24_) assay, surface tension reduction, oil spreading, and BATH assay were performed to determine biosurfactant production. E_24_ tests were performed for different values of temperature, pH and salt concentration to determine the ideal conditions for emulsion stability. For identification of the nature of biosurfactant, chromatographic studies were performed and the biosurfactant was chemically characterized using LCMS, GCMS, NMR and FTIR.

## Materials and methods

*Gordonia* sp. IITR100 (MCC 2877), a biodesulfurizing bacterium previously isolated in our lab from a petroleum-contaminated soil sample using an enrichment culture, was used in the present study [33].

### Screening for biosurfactant production

*Gordonia* sp. IITR100 was screened for its ability to produce biosurfactant by five different methods. The isolated strain was inoculated in 200 ml of Luria broth by transferring 1% of the overnight grown seed culture (containing 2.3 *10^6^ CFU/ml) into the flask. The flask was incubated in a rotatory shaker for 7 days at 30°C and 180 rpm. The flasks were harvested by centrifuging the culture at 8400 g and 4°C for 30 min. The supernatant (cell-free culture) was collected and used for performing biosurfactant screening assays, emulsion stability assays and surface tension reduction measurement. Assays performed to determine biosurfactant production include: drop collapse assay, oil spreading assay, microplate assay, emulsion index assay and surface tension measurements. The experiment was repeated twice in triplicates.

### Drop-collapse assay

Drop-collapse assay was performed in polystyrene 48-well microplates and on glass slides, as illustrated by Mohanram et al [27]. Wells and glass slides were coated by addition of 20 μl of burnt engine oil followed by 10 μl of culture supernatant (cell-free extract) or controls added to the centre of the oil. The drop added was left undisturbed for 2–3 min and its collapse was monitored. If the drop collapsed, the supernatant was scored positive for the presence of biosurfactant. Approximately 10% SDS was taken as positive control and water was taken as negative control. Tests were repeated 5 times.

### Oil spreading assay

The oil spreading assay was carried out as illustrated by Youssef et al [36]. Distilled water (25 ml) was poured in a petri dish. One hundred microlitre of used engine oil was poured carefully on the surface of the water. Ten microlitre of culture supernatant (cell-free extract) was added to the centre of the surface of petrol and the formation of a clearing zone as a result of displacement of petrol was monitored. Tests were conducted in triplicate.

### Microplate assay

The microplate assay was performed as illustrated by Vaux and Cottingham 2007 [37]. One hundred microlitres of supernatant (cell-free extract) were added to a well of a 48-well microplate and the plate was viewed against the backing sheet containing grids. Optical distortion of background grids was monitored and the assay was scored positive if distortion was observed. This optical grid distortion provides qualitative assessment for detecting the presence of biosurfactant(s).

### Emulsion index (E_24_)

The stable emulsion index (E_24_) was determined as illustrated by Mohanram et al [27]. Equal volume (2.5 ml) of cell-free supernatant and petrol was added to a test tube. The resulting mixture was vortexed vigorously for 3 min and left undisturbed overnight. After 24 hours, the stable emulsion index (E_24_) was determined as the percentage of height of the emulsified layer (cm) divided by height of the entire liquid column (cm). The test was repeated with diesel, mineral oil and hexadecane in place of petrol. Approximately 10% SDS was taken as positive control and water was taken negative control. Tests were performed in triplicate.

In all the biosurfactant screening assays, SDS (10%) and cell-free extracts from other biosurfactant-producing microbes (*Rhodococcus* sp. IITD102 and *Paenibacillus* sp. IITD108) were taken as positive control and water was taken as negative control.

### Surface tension measurements

To confirm the production of surfactant, surface tension of the supernatant (cell-free broth) was measured and compared to that of the control (autoclaved uninoculated medium). A decrease in surface tension of cell free supernatant due to the growth of microorganisms would confirm the production of biosurfactant. A digital K12 –Kruss tensiometer, Germany, was used for determining the surface tension of the cell-free broth and the control medium. Surface tension was measured using Du Noüy ring method (ASTM D1331). Here also, cell-free extracts from other biosurfactant-producing microbes (*Rhodococcus* sp. IITD102 and *Paenibacillus* sp. IITD108) were taken as positive control and uninoculated culture medium was taken as negative control.

### Confocal microscopy of the emulsion

A confocal microscope (Leica TCS SP8 Confocal Laser Scanning Microscope) was used to examine the microstructure of the W/O emulsion droplets. Before analysis, the emulsions were gently stirred to ensure a homogenous sample. Approximately 20 μl of the samples were placed on the glass slide, and a cover slip (0.17 mm thickness) was placed on top to ensure that no air gaps (or bubbles) were caught between the sample and the coverslip. The samples were scanned using a 20X objective lens at room temperature (25°C). Image acquisition was performed using LAS X application software.

### Biosurfactant extraction

The cell-free broth after harvesting the culture was collected separately and used for extraction of surfactant. For biosurfactant extraction, the pH of the cell-free broth was adjusted to 2 using 11.5 N HCl solution and was stored overnight at 4°C. Surfactant was extracted by using a solution of chloroform and methanol in the ratio 2:1 [38,39]. Chloroform-methanol mixture was added to cell-free broth in equal volume and mixed vigorously for 10 min. The mixture was then left undisturbed for 20 min and allowed to settle, after which the upper phase (aqueous phase/methanol phase) was pipetted into a fresh beaker. The lower phase containing the surfactant was left to evaporate at room temperature inside a fume hood. Extraction was repeated three times. Honey coloured crude biosurfactant was recovered from the bottom of the beakers using 5 ml of the 2:1 chloroform and methanol mixture. This solution was concentrated to 3 ml and used for chromatographic analysis and identification of biosurfactant.

### Emulsion stability studies

Emulsion stability assays were performed as described by Campos et al. to determine the ideal conditions for stability and functioning of biosurfactant produced by *Gordonia* sp. IITR100 [40]. Emulsion stability at different temperatures, pH and salt concentrations was determined by performing E_24_ assay (with petrol) under each condition.

The effect of temperature on the stability of emulsion was determined by performing E_24_ assay of cell-free culture with petrol and incubating the emulsion formed at 4°C, 20°C, 30°C, 37°C, 50°C and 60°C. The effect of pH and salinity on the stability of emulsion was determined by adjusting the pH (using 1 N HCl or 1 N NaOH) or salt concentration (by addition of NaCl) of the cell-free broth to the desired value and calculating E_24_ indices after 24 hours. The pH was varied from 2–10 (2, 4, 6, 7, 8 and 10) and salt concentration from 0–8% (0%, 0.5%, 1%, 2%, 4%, 6% and 8%).

### BATH assay

The Bacterial adherence to Hydrocarbons (BATH) assay was performed as illustrated by Mohanram et al. 2016 with slight modifications. Flask containing 100 ml of Luria Broth was inoculated by transferring 1% of overnight grown seed culture (containing 3.2 *10^6^ CFU/ml) of *Gordonia* sp. IITR100. The flasks were incubated at 30°C with constant shaking at 180 RPM for 3 days to reach its logarithmic phase. The cells were then harvested at 8400 g at 4°C for 30 min. The supernatant was collected separately while the cells were resuspended in 25 ml of phosphate urea magnesium sulfate (PUM) buffer. The composition of PUM buffer was 16.948 g/l of K_2_HPO_4_, 1.8 g/l of urea, 7.26 g/l of KH_2_PO_4_ and 0.2 g/l of MgSO_4_^.^7H_2_O. To 6 ml bacterial suspension, 3 ml of petrol was added in a test tube. The test tube was pre-incubated at 30°C for 10 min, after which it was vortexed for 5 min and left to settle for 45 min. The 2 phases were allowed to separate. Using a spectrophotometer, the optical density (OD) of the aqueous phase before (OD_1_) and after petrol treatment (OD_2_) was determined at 400 nm. The values were expressed in terms of percentage of bacterial cells adhering to petrol as compared to the control (suspension without addition of petrol). The test was repeated with petrol in place of hexadecane.

%BATH = (1−(OD_2_/OD_1_)) _X_ 100.

### Determination of critical micelle concentration (CMC)

CMC of a biosurfactant is the concentration at which micelles begin to form and surface tension reaches its lowest value. To evaluate the CMC of the biosurfactant, dried extracted biosurfactant was dissolved in milliQ water at various concentrations ranging from 10 mg/l to 500 mg/l, and the surface tension of the resulting mixtures was measured using a digital K12 –Kruss tensiometer and the Du Noüy ring method. The biosurfactant’s CMC value was determined as the inflection point of the surface tension versus biosurfactant concentration curve.

### Kinetics of biosurfactant production

Kinetic studies on biosurfactant production were performed in a stirred tank bench top reactor (Applikon) with a tank diameter of 0.16 m and working volume of 3.5 l. The vessel was equipped with two Rushton turbine impellers of diameter 0.06 m. The composition of the culture medium used for the growth of the microbial strain and for studying the kinetics of the biosurfactant production was: 2.00 g/l Na_2_HPO_4_, 1.00 g/l KH_2_PO_4_, 4.25 g/l ammonium oxalate, 0.40 g/l MgCl_2_, 0.025 g/l ammonium sulphate and 17 g/l sucrose. The medium was supplemented with trace elements (1 ml/l) of composition: 0.05 g/l KI, 0.05 g/l LiCl, 0.80 g/l MnCl_2_.4H_2_O, 0.50 g/l H_3_BO_3_, 0.10 g/l ZnCl_2_, 0.10 g/l CoCl_2_.6H_2_O, 0.10 g/l NiCl_2_.6H_2_O, 0.05 g/l BaCl_2_, 0.05 g/l (NH_4_)_6_Mo7O_24_.2H_2_O, 0.50 g/l SnCl_2_.2H_2_O and 0.10 g/l Al(OH)_3_. Culture medium was prepared and added to the vessel. The component parts were mounted on the vessel and the entire reactor was sterilized by autoclaving at 121°C for 15 mins. After cooling down, the reactor was inoculated with 1% seed culture (containing 1.5 *10^7^ CFU/ml) and operated at 30°C and 350 rpm till the stationary phase was reached. The pH of the broth was maintained at 7 by addition of 2 M NaOH or 2 M HCl in response to the change in pH value. During the course of the reactor run, regular liquid sampling was performed during which 20 ml of the sample was withdrawn from the reactor. Biomass growth was determined by measuring the light absorbance of the liquid samples at a wavelength of 600 nm. The collected sample was centrifuged at 8400 g for 15 min at 4°C and the cell free supernatant was used for extraction of the biosurfactant, determination of emulsion index and determination of the sugar uptake. HPLC equipped with a refractive index detector (Agilent, USA) was performed for detection of sucrose uptake as described by Zargar et al. [41].

### Characterization of biosurfactant by thin layer chromatography

To determine the nature of the biosurfactant, approximately 4 μl of extracted biosurfactant was loaded onto a TLC plate. TLC was performed using methanol as mobile phase in a saturated chamber. After completion of the run, TLC plates were analysed using UV and developed using iodine, ninhydrin, anthrone and anisaldehyde. The plates were taken out and R_f_ values were calculated.

Iodine vapor staining was performed to detect the presence of lipids [42]. A glass chamber was saturated with iodine (I_2_) vapours using iodine crystals. The TLC plate was placed inside the saturated chamber and covered with a glass plate. The appearance of yellow-brown spots due to reversible reaction of iodine vapours with lipids was monitored.

Ninhydrin test was performed to detect the presence of peptides and amino acids. One percent (100 mg/ml) solution of glycine was used as positive control and 1% solution of glucose was used as negative control. Ninhydrin stain was prepared by adding 0.4 g of ninhydrin in 20 ml butanol and 0.6 ml acetic acid. This solution was sprayed on the TLC plate. The plate was then transferred to an incubator at 110°C and checked after every 5 minutes for development of any coloured spots.

Anthrone test was performed to detect the presence of sugars [43]. One percent (100mg/ml) solution of glucose was used as positive control and 1% solution of glycine was used as negative control. Anthrone stain was prepared by adding 0.2 g of anthrone in 5 ml H_2_O and 95 ml H_2_SO_4_. This solution was sprayed on the TLC plate. The plate was then transferred to an incubator at 110°C and checked after every 5 minutes for development of green or greenish-blue spots.

Anisaldehyde test was performed to detect the presence of sugars that usually contain nucleophiles such as alcohols, ketones or aldehydes. One percent (100 mg/ml) solution of glucose was used as positive control and 1% solution of glycine was used as negative control. p-Anisaldehyde stain was prepared by adding 2 ml p-Anisaldehyde in 1 ml H_2_SO_4_ and 48 ml acetic acid. This solution was sprayed on the TLC plate. The plate was then transferred to an incubator at 110°C and checked every 5 minutes for development of any coloured spots. Commercial rhamnolipid from AGAE Technologies, USA (Lot No. A79212328003) was used as a positive control during TLC characterization of the biosurfactant.

Purification of the crude biosurfactant was performed using a silica column as suggested by Zargar et al. [41].

### Chemical identification of biosurfactant

Biosurfactant was identified by performing liquid chromatography mass spectrometry (LCMS) using LCMS Agilent Technologies 1260 Infinity LC equipped with 6410 Triple Quad MS, USA and ZORBAX C18 column. Two microlitre of extracted biosurfactant was injected into the column. Chloroform/methanol mixture (3:1) at a flowrate 0.1 ml/min was used as the mobile phase. Detection time of 20 min was set in which m/z ranges from 100 to 1200 were scanned. LCMS was performed in positive electrospray (ES) mode and the m/z values obtained were analysed using METLIN.

Further characterization of the extracted biosurfactant was done by performing gas chromatography mass spectrometry (GCMS) analysis. For GCMS, 10 μl sample was injected into the column. For identification of the biosurfactant, ions were analysed and identified using 5977B GC-MSD gas chromatography mass spectrometer (Agilent). The injection temperature was maintained at 260°C. The oven temperature was programmed to start at 40°C, which was held for 2 min, and then ramped to 280°C at a rate of 6°C/min and finally to 280°C at a rate of 10°C/min. MS source and MS Quad temperatures were maintained at 230°C and 150°C.

To determine the functional groups and the type of bonding present, Fourier-transform infrared spectroscopy (FTIR) of the extracted biosurfactant was performed using FTIR Thermo Fisher Scientific Nicolet iS50 in ATR mode between 400 and 4000 cm^-1^.

To further characterize and confirm the structure of the extracted biosurfactant, H^1^ and C^13^ nuclear magnetic resonance (NMR) was performed using Bruker Avance AV-III type spectrometer. For H^1^ NMR, 5 mg of extracted biosurfactant was dissolved in 1 ml of CDCl_3_ while in C^13^, 30 mg of sample was dissolved in 0.5 ml of CDCl_3_. The spectrum was recorded at 297.9 K at a frequency of 500 MHz.

### Bioinformatic analysis of the genome

Bioinformatic analysis of the genome was performed to check for the presence of genes involved in the synthesis of glycolipid biosurfactants. The microbial pathways for the biosynthesis of glycolipid biosurfactants (especially rhamnolipids) have been extensively studied and the formation of rhamnolipids has been found to occur in 3 steps [44,45]:

1. Synthesis of fatty acid moiety (Hydroxy acyl-ACP) from acyl-CoA.
2. Conversion of dTDP D-glucose to dTDP L-rhamnose.
3. Coupling of dTDP L-rhamnose with hydroxyalkanoyloxy alkanoic acid to form rhamnolipid.

A similar mechanism for the biosynthesis of other glycolipids may occur in *Gordonia* sp. IITR100. Genes involved in the biosynthesis of C18 fatty acid chains which form the hydrophobic part of the biosurfactant produced by *Gordonia* sp. IITR100 were studied using KEGG pathways. The pathway was specified for different species of *Gordonia*, including *Gordonia terrae* and *Gordonia alkanivorans*. Similarly, genes involved in the synthesis of L-rhamnose and other carbohydrate moieties of biosurfactants, as well as those involved in linking carbohydrate moieties to lipid chains to form glycolipid biosurfactants, were studied using KEGG pathways and previous literature reports. Then the genome of *Gordonia* sp. IITR100 was screened for the presence of these genes to confirm the pathway for biosynthesis of the identified biosurfactant.

## Results

### Biosurfactant production assays

**Fig 1:**
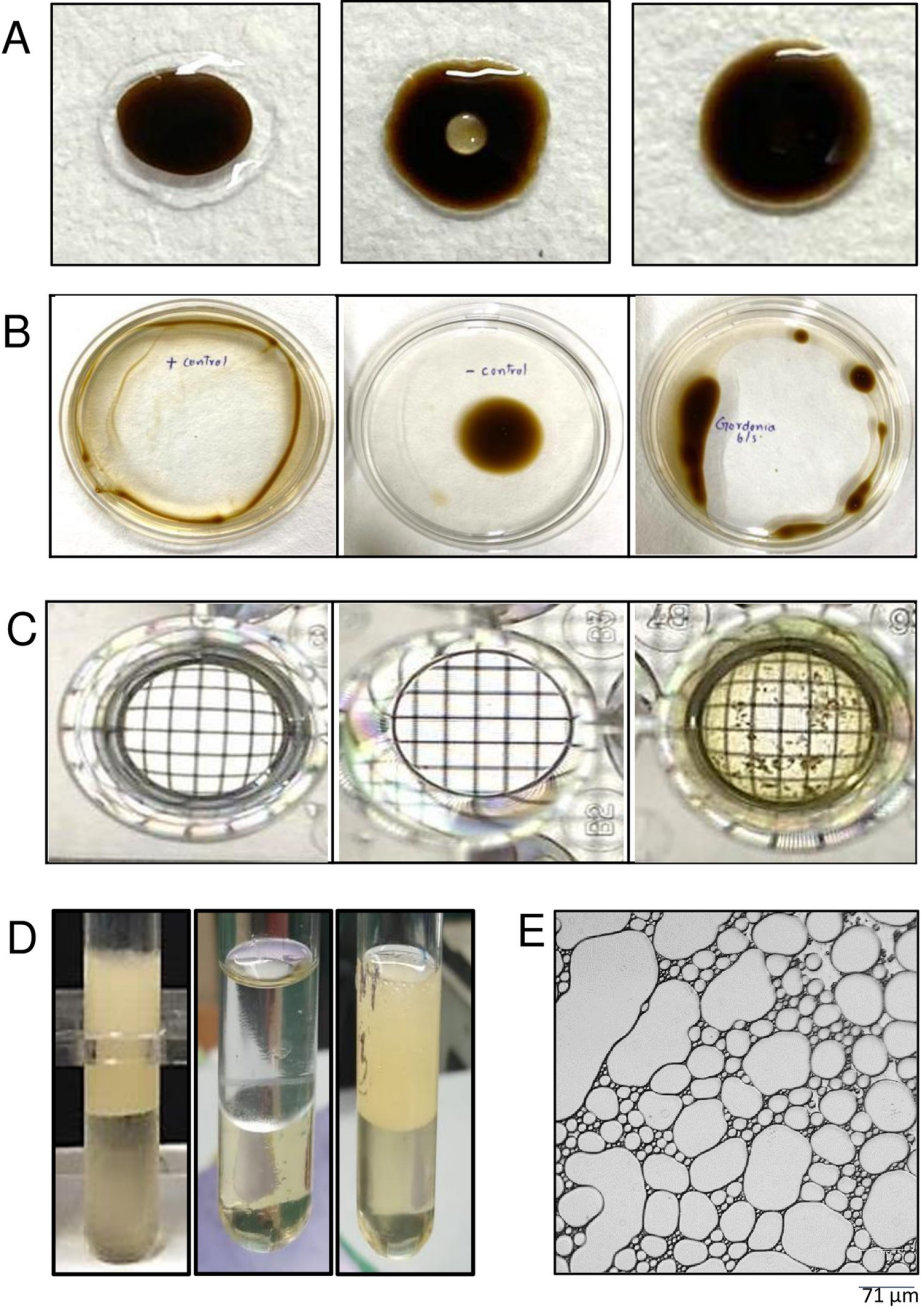
Biosurfactant screening assays: A. Drop collapse assay on glass slide, B. Oil spreading assay, C. Microplate assay, D. Emulsion index assay, E. Microscopic image of the emulsion formed by cell free supernatant of *Gordonia* sp. IITR100. (Left panel) 10% SDS (Middle panel) water, and (Right panel) cell free extract of *Gordonia* sp. IITR100.

All the biosurfactant screening assays gave positive results. In the drop collapse assay (Fig 1A), drop collapse was observed after addition of cell-free culture and 10% SDS and other positive controls (cell-free extracts of *Rhodococcus* sp. IITD102 and *Paenibacillus* sp. IITD108) while no drop collapse was observed when water (negative control) was added to the oil-coated well. In the oil spreading assay, a clear zone of displaced oil was formed when crude biosurfactant-containing cell-free broths from *Gordonia* sp. IITR100, *Rhodococcus* sp. IITD102 and *Paenibacillus* sp. IITD108 were added with 10% SDS, while no oil spreading was observed for water (Fig 1B). In the microplate assay, grid distortion was observed for the crude biosurfactant from *Gordonia* sp. IITR100 and positive controls, while no grid distortion was observed in the case of water (Fig 1C). E_24_ assay also showed the production of biosurfactant with an emulsion index of approximately 47% with petrol (Fig 1D) and 52.2% with mineral oil. An emulsion index of 38.9% was obtained when diesel was used in the study. Microscopic imaging of the emulsion showed that emulsion formed was water in oil microemulsion. The droplets of size > 50 μm correspond to oil droplets while the droplets of size <15 μm are water droplets.

Biosurfactant production was confirmed by determining the surface tension of the culture supernatant and comparing it with that of the control (uninoculated medium). The surface tension of the milliQ water and the control medium were found to be 72 mN/m and 61.07 mN/m, respectively, and that of the culture supernatant was found to be 36.82 mN/m. Therefore, the biosurfactant was successful in reducing the surface tension of the medium by 40%. This decrease in the surface tension of the medium served as a confirmatory test for the production of biosurfactant by the bacteria. *Rhodococcus* sp. IITD102 and *Paenibacillus* sp. IITD108 were successful in reducing the surface tension of the culture medium to 41.41 mN/m and 28.43 mN/m, respectively.

Crude biosurfactant at a concentration of 4.02 g/l was produced by *Gordonia* sp. IITR100 during growth in Luria broth.

### Emulsion stability studies

**Fig 2:**
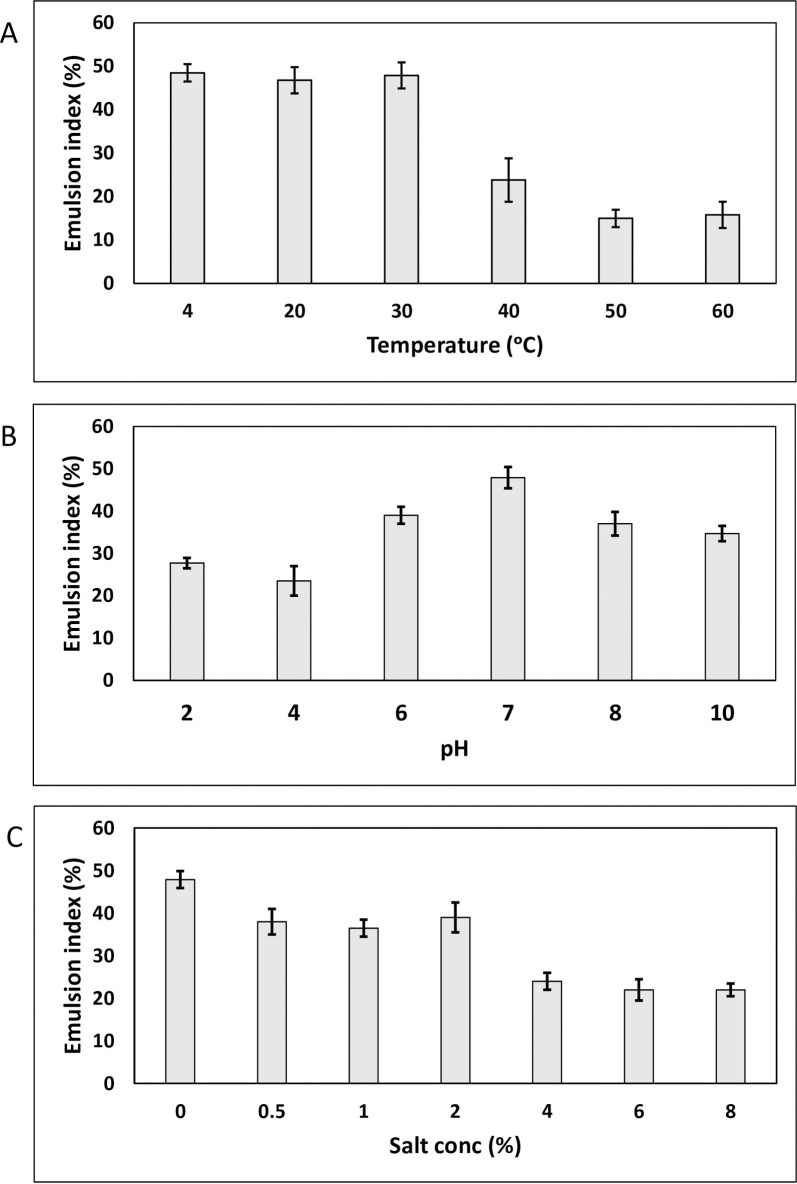
Emulsion stability studies: Effect of temperature (A), pH (B) and salt concentration (C) on emulsion stability. Statistical analysis of the results was performed using single factor ANOVA and p-values for temperature, pH and salt concentration profiles < 0.0001.

**Fig 3:**
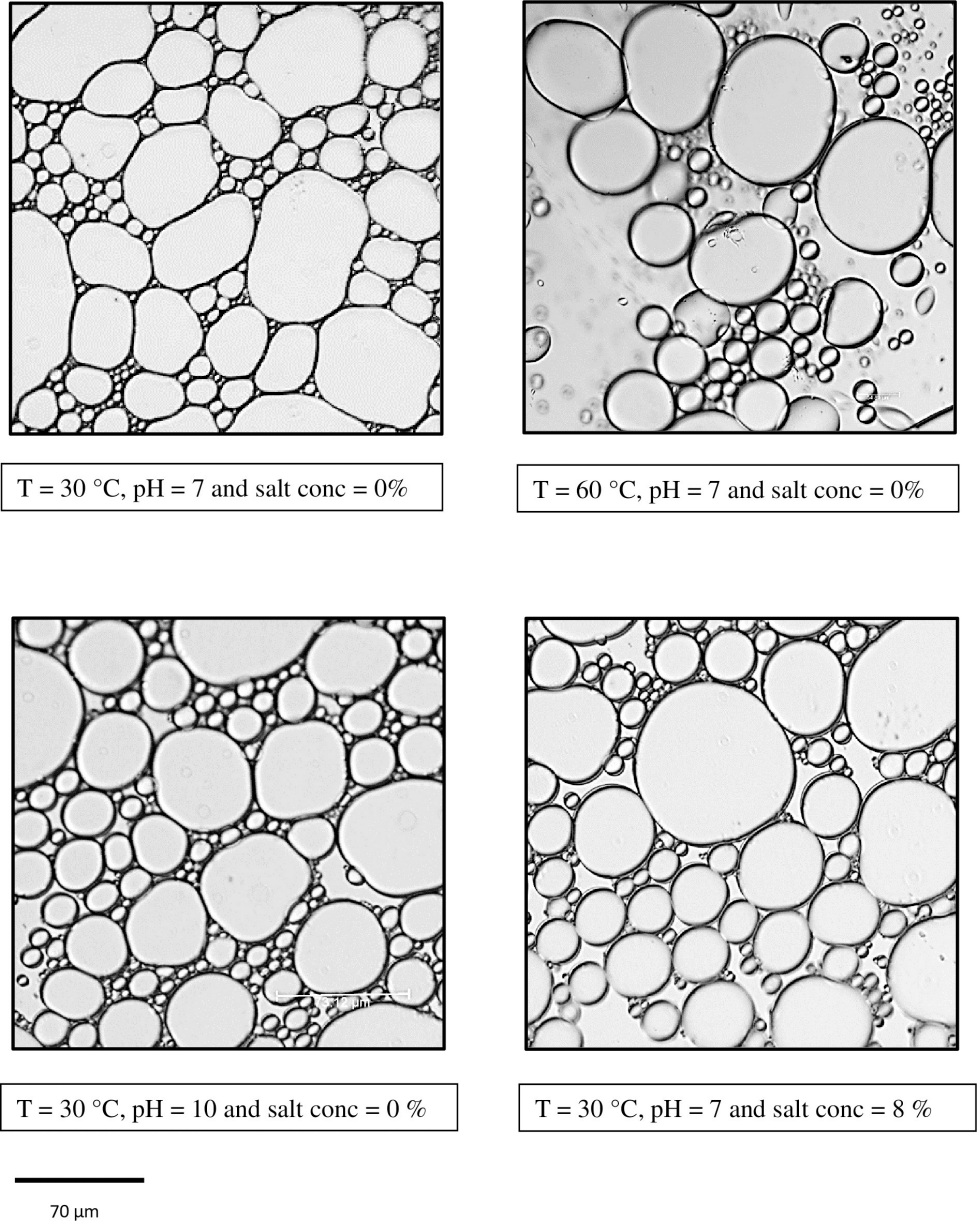
Effect of temperature, pH and salt concentration on the microstructure of emulsion.

Emulsion stability studies revealed that the emulsion was stable over a temperature range of 4°C to 30°C (Fig 2A). At 40°C the emulsion index reduced from 47.9 to 23.8% which further reduced to 15% at 50°C and 60°C. Microscopic imaging of the emulsion showed that at higher temperature (60°C), the number of water droplets trapped between oil droplets was significantly lower than that at lower temperature (30°C) (Fig 3). Emulsion stability was found to be minimum under acidic conditions. Maximum stability was obtained at neutral pH. Under alkaline conditions, the emulsion was found to be relatively stable with the emulsion index decreasing from 47.9% at pH 7 to 34.7% at pH 10 (Fig 2B). Salt concentration higher than 2% was found to decrease the stability of the emulsion with the emulsion index reducing from 47.9% at 0% salt concentration to 22% at 4 to 8% salt concentration (Fig 2C). Salt concentration and pH did not have a significant effect on the microstructure of the emulsions formed (Fig 3). Therefore, the ideal conditions for stability and function of biosurfactant produced by *Gordonia* sp. IITR100 are temperatures up to 30°C, pH of 7 and salt concentration below 2%.

### BATH assay

Cell surface hydrophobicity was determined with petrol and hexadecane. The bacterial strain showed lower adherence to hydrocarbons. Percentage BATH with petrol and hexadecane were found to be 13.44% and 18.7%, respectively. This indicates that the bacterial surfaces are hydrophilic in nature and are not responsible for the emulsion formation. Therefore, emulsion formation is a result of biosurfactant production and not surface characteristics of the bacterial cell.

### Determination of critical micelle concentration (CMC)

**Fig 4:**
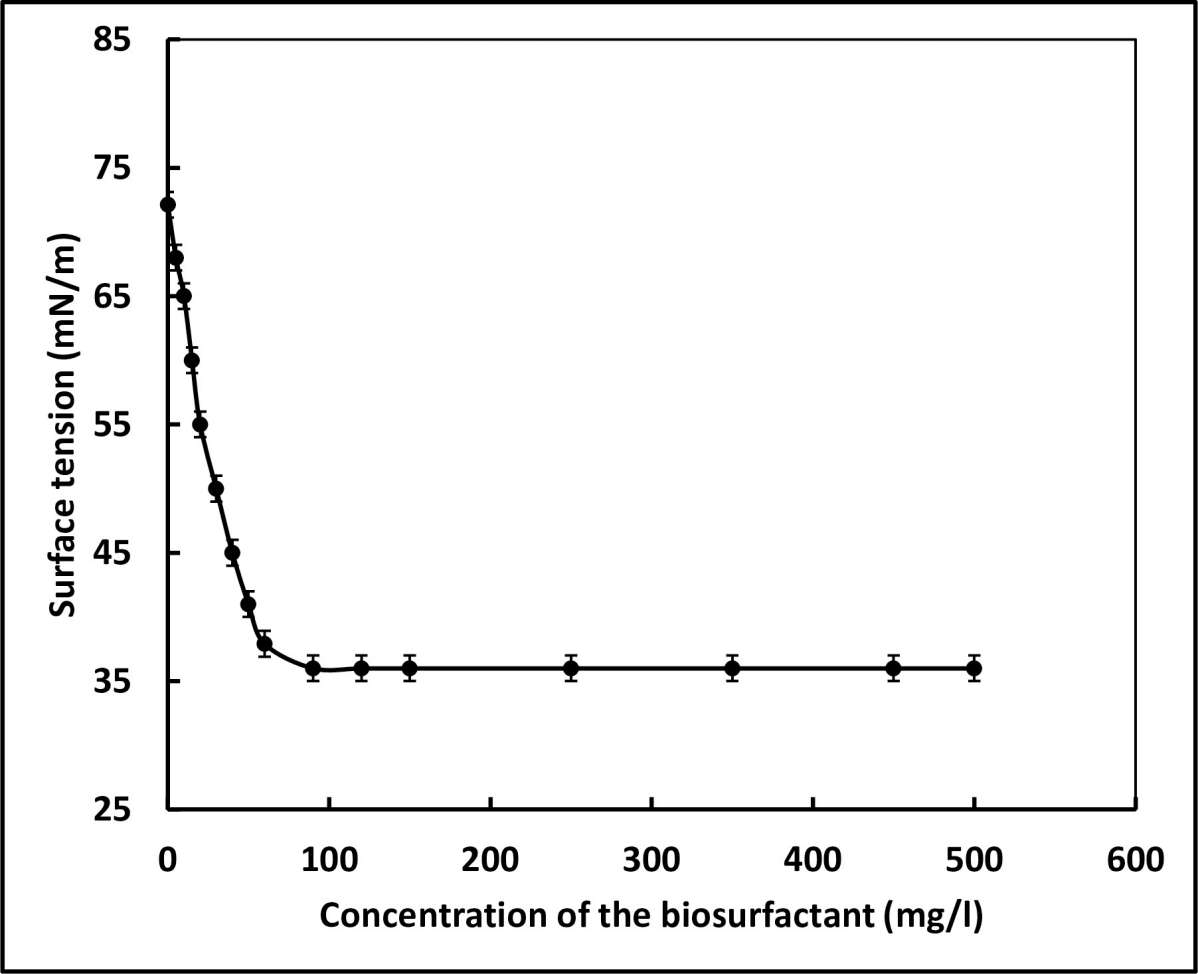
Critical micelle concentration of the biosurfactant.

The concentration of the biosurfactant at which the lowest value of surface tension is achieved is referred to as the CMC. The surface tension of pure milliQ water (free of biosurfactants) was measured to be 72 mN/m. Addition of the biosurfactant to milliQ resulted in the decrease in its surface tension. The surface tension of water decreased as a function of the concentration of biosurfactant added to it and reached a minimum of 36 mN/m when 90 mg/l of biosurfactant was added to it (Fig 4). As the concentration of the biosurfactant was further increased, no further change in the surface tension was observed suggesting that the CMC of the biosurfactant is 90 mg/l.

### Kinetics of biosurfactant production

**Fig 5:**
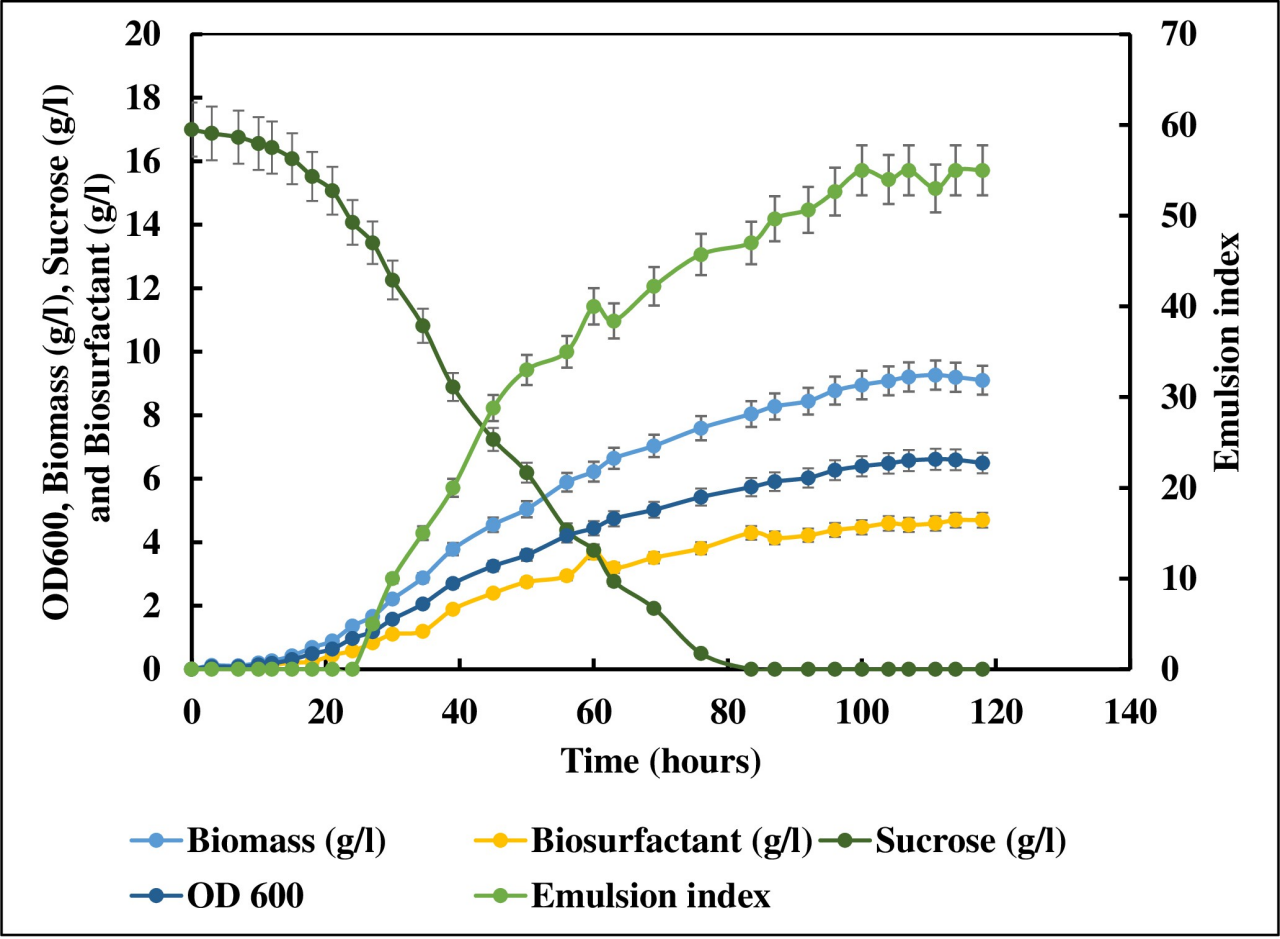
Biosurfactant production in a 5 L bioreactor.

Kinetics of the biosurfactant production was studied in a 5 l batch reactor with a working volume of 3.5 l (Fig 5). After inoculation, a lag phase of 16 hours was observed during which no biosurfactant production was observed. Log phase was observed after 16^th^ hour as the microbes started growing exponentially till the culture reached an OD_600_ of 6.5 (biomass concentration of 9.2 g/l). During the exponential growth phase, sucrose was rapidly utilized by the growing culture and completely consumed after 80 hours of batch run. This marked the start of the stationary phase. Biosurfactant production was found to be growth associated since the microbes started producing the biosurfactant during early growth phase and reached a maximum concentration of 4.7 g/l at the end of exponential phase. Stationary phase was observed after 90^th^ hour of the run and no further biosurfactant production was observed during this phase. At the end of the fermentation run, an average dry biomass concentration of 9.2 g/l and biosurfactant concentration of 4.7 g/l were obtained. The yield of biomass (Y_X/S_) and biosurfactant (Y_P/S_) with respect to sucrose were 0.54 g/g and 0.27 g/g respectively. During the exponential phase biosurfactant was produced at a rate 0.07 g/lh while biomass was produced at a rate 0.14 g/lh.

### Characterization of biosurfactant

**Fig 6:**
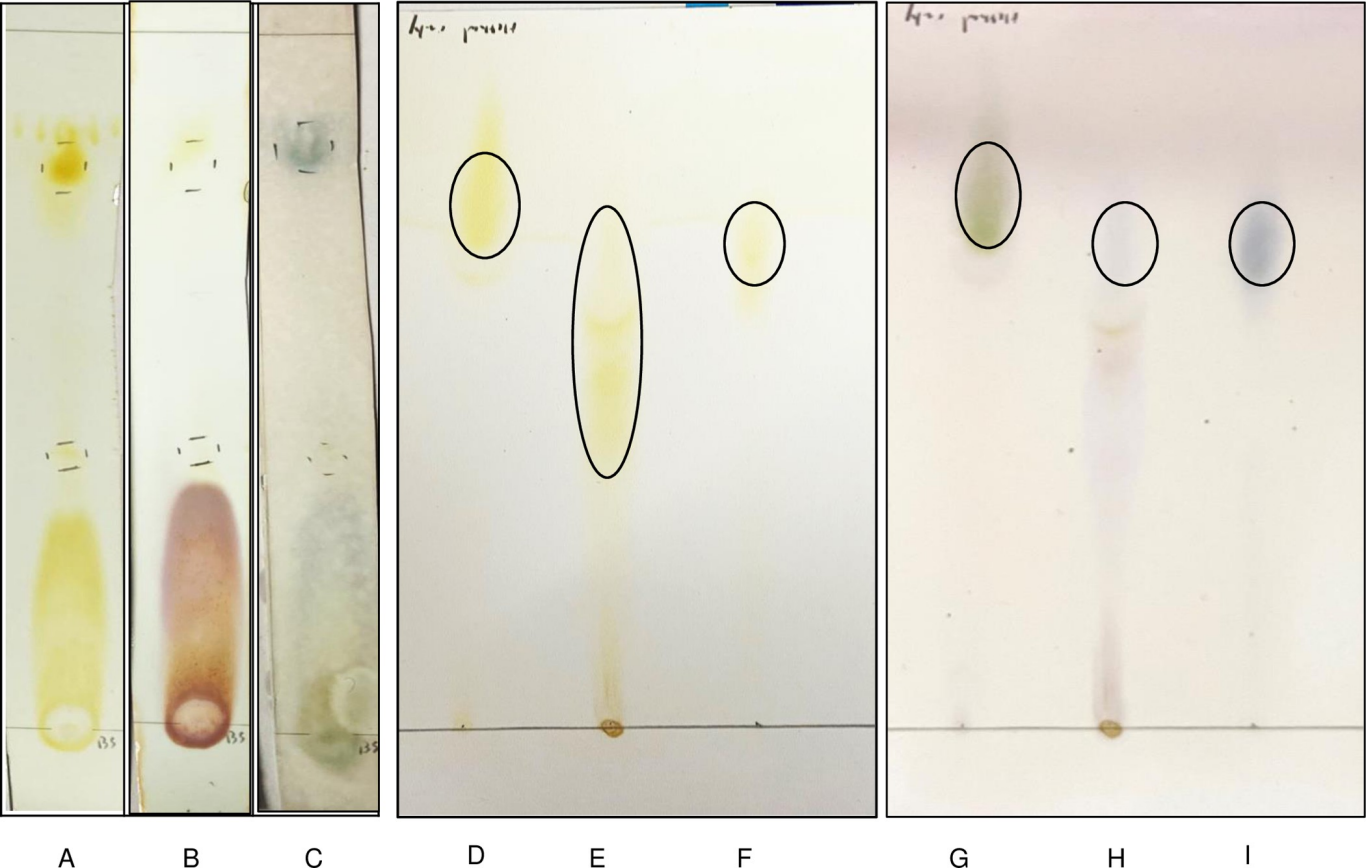
TLC characterization of biosurfactant. Left panel: Crude biosurfactant (A) stained with iodine (B) stained with ninhydrin (C) stained with p-anisaldehyde. Middle panel: Iodine staining for lipid detection (D) control rhamnolipid (E) crude biosurfactant (F) purified biosurfactant. Right panel: p-anisaldehyde staining for carbohydrate detection (G) control rhamnolipid (H) crude biosurfactant (I) purified biosurfactant.

Staining the TLC plate with iodine vapor resulted in the development of yellow spots as soon as it was placed in the iodine-saturated chamber (Fig 6A and 6E), showing the presence of lipid in the crude biosurfactant. Staining of TLC plate with ninhydrin did not result in any blue colour development (Fig 6B), showing that the crude biosurfactant does not contain any peptide or amino acid. The presence of a carbohydrate moiety in the crude biosurfactant was confirmed by development of light blue spots when the TLC plate was stained with anisaldehyde reagent (Fig 6C), Therefore, the crude biosurfactant contains a lipid part and a carbohydrate part and, as such, is glycolipid in nature.

Chromatographic analysis of the purified biosurfactant showed a single spot on a TLC which confirmed the removal of impurities from the biosurfactant mixture (Fig 6F and 6I). The control rhamnolipid (Fig 6D and 6G) and the purified biosurfactant (Fig 6F and 6I) both stained positive for the presence of lipid and the carbohydrate and showed similar Rf values. Rf value for the commercial rhamnolipid (control) was 0.74 while that for the biosurfactant produced by *Gordonia* sp. IITR100 was found to be 0.70. This confirmed that the purified biosurfactant is a glycolipid.

### Chemical identification of the biosurfactant

**Fig 7:**
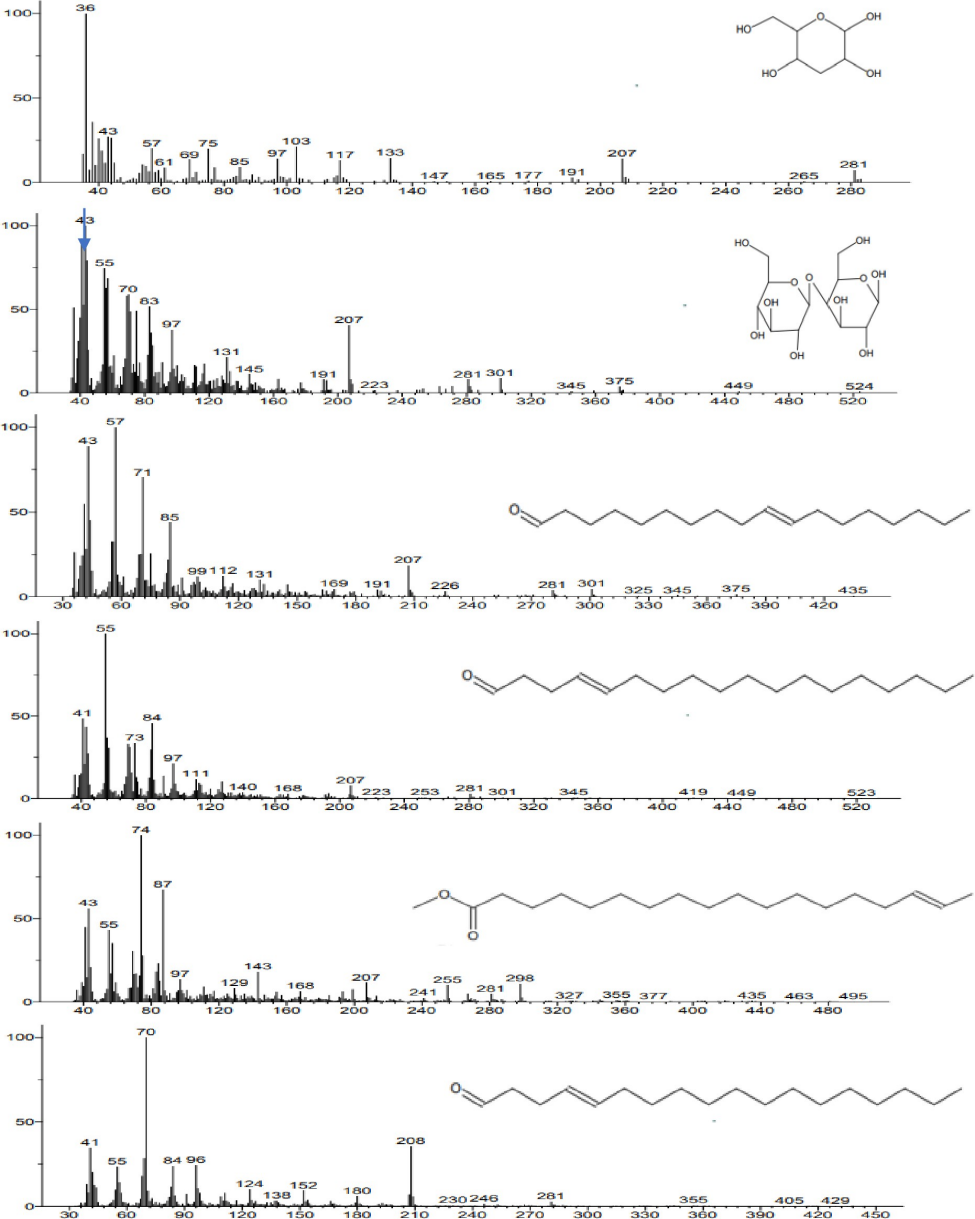
GC-MS spectrum of the extracted biosurfactant.

**Fig 8:**
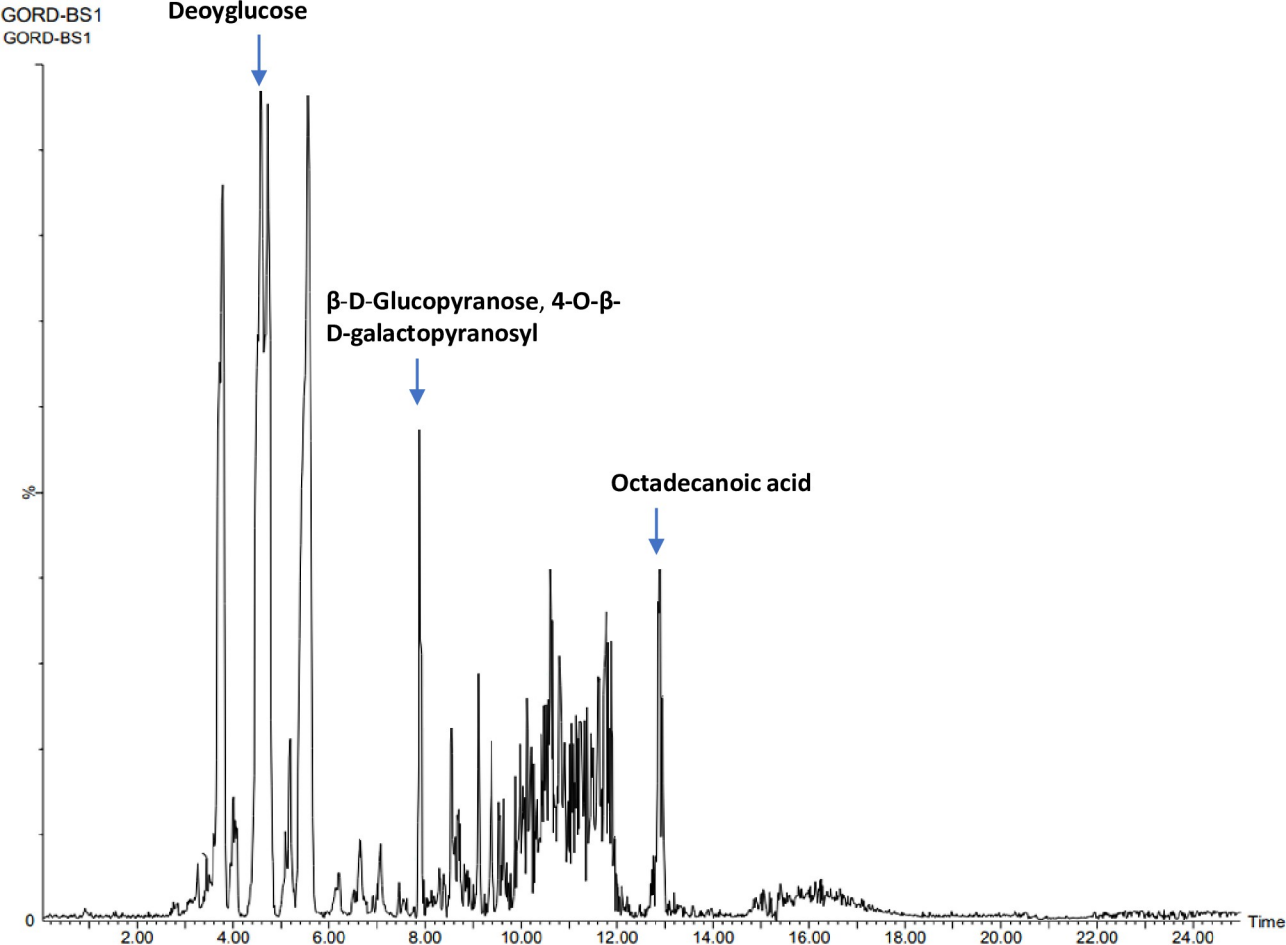
GC-MS chromatogram.

LCMS data obtained was analysed using METLIN software at an accuracy of 10 ppm. The biosurfactant was identified as a glycolipid containing a hydrophobic tail of 18C octadecanoic acid (molecular weight 285 g/mol) and β-D-glucopyranose, 4-O- β-D-glucopyranosyl (345 g/mol) as a hydrophilic moiety. In LCMS these fragments were detected at a ppm accuracy of 9 and 6, respectively. In the GCMS spectrum (Fig 7), fragments of biosurfactant, octadecanoic acid methyl ester (m/z value 296) and β-D-glucopyranose, 4-O- β-D-glucopyranosyl (m/z value 345) were detected at retention time of 12.9 min and 7.9 min, respectively (Fig 8). MS breakdown fragments of both parts were also detected in LCMS and GCMS spectra e.g., in GCMS, an analysis peak for 3-deoxyglucose (m/z value 164.16) was obtained at a retention time of 4.58 min, while the peak for octadecanal (m/z value 268) was obtained at retention time of 12.9 min (Fig 8). From LCMS and GCMS studies, the overall molecular weight of the biosurfactant was estimated to be approximately 630 g/mol.

**Fig 9:**
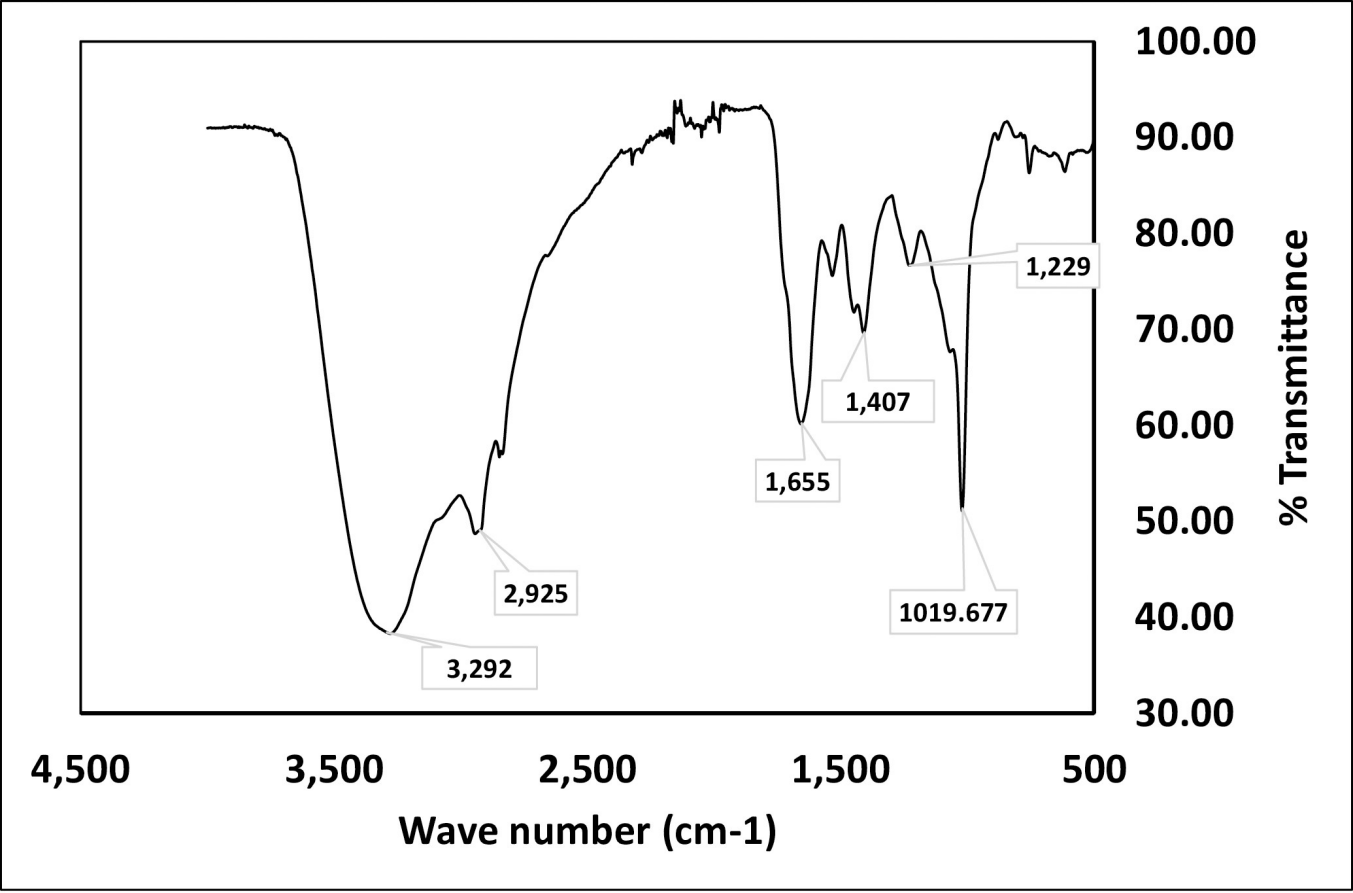
FTIR of biosurfactant produced by *Gordonia* sp. IITR 100.

FTIR analysis of the extracted biosurfactant (Fig 9) showed a strong peak at 3292 cm^-1^ which corresponds to OH stretching. Small peaks at 2933 cm^-1^ and 2832 cm^-1^ were obtained, these designate the presence of CH bonds. Peaks corresponding to C-O were obtained at wave numbers 1218 cm^-1^ and 1020 cm^-1^. Another peak corresponding to C = O was obtained at 1649 cm^-1^. This spectrum perfectly matches the compound identified through LCMS analysis.

**Fig 10:**
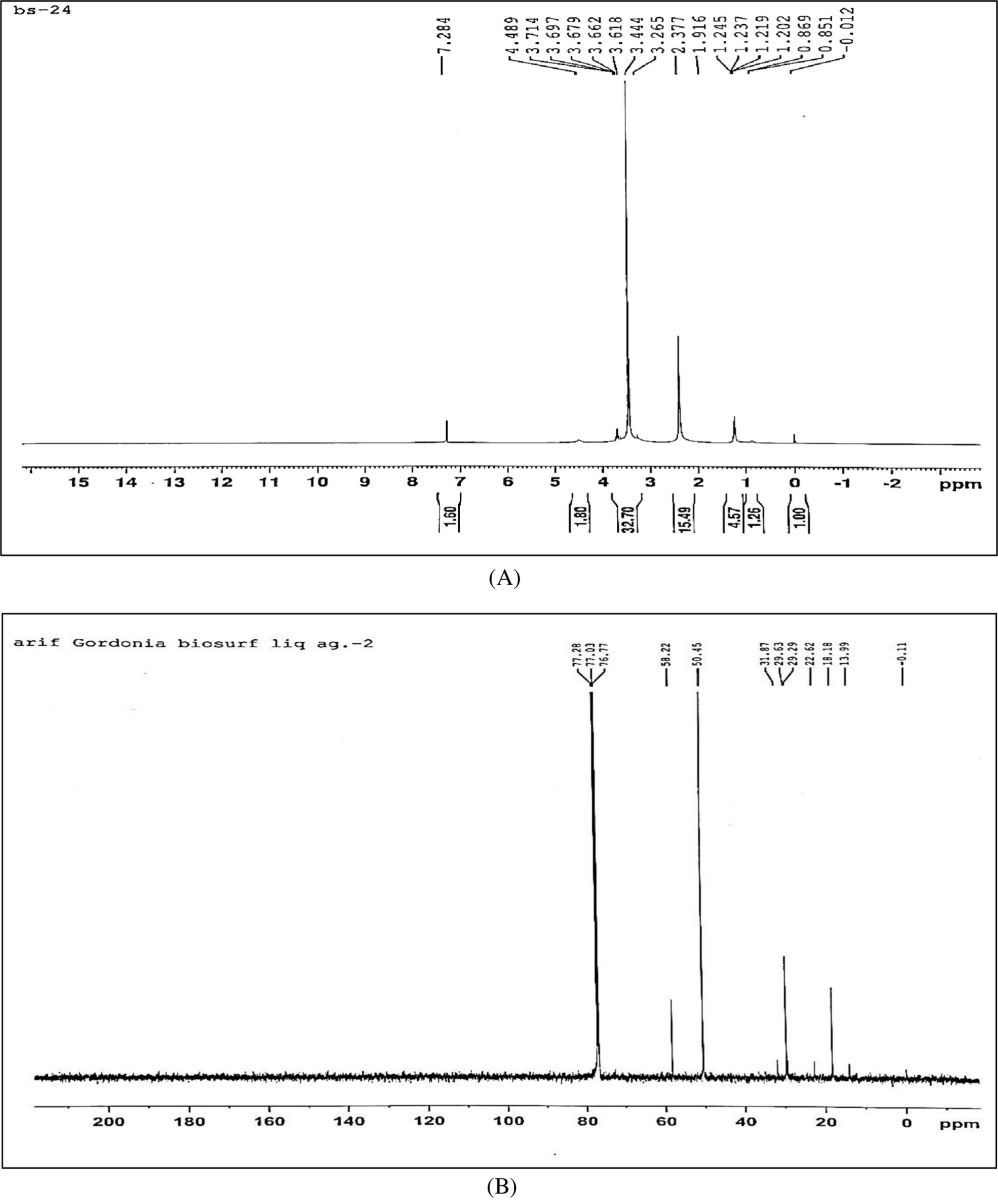
NMR of biosurfactant produced by *Gordonia* sp. IITR 100. (A) H^1^ NMR (B) C^13^ NMR.

H^1^ NMR of the extracted biosurfactant (Fig 10A) showed peaks at chemical shifts 0.8 which confirmed the presence of methyl hydrogens (R-CH_3_). A peak obtained at 1.1 shows the presence of alkyl hydrogens (R-CH_2_-R). Peaks obtained in the range 3.1–3.7 confirm the presence of hydrogens associated with a C-O bond. Peaks within the range 3.0 to 3.7 also correspond to hydrogens of alcohol and ester bonds.

C^13^ NMR of the extracted biosurfactant (Fig 10B) showed peaks at chemical shift 13.9 which correspond to C of R-CH_3_. Peaks between 18 to 30 designate C of R-CH_2_-R. Carbon attached to oxygen is designated by peaks between shifts 50 to 78 ppm.

**Fig 11:**
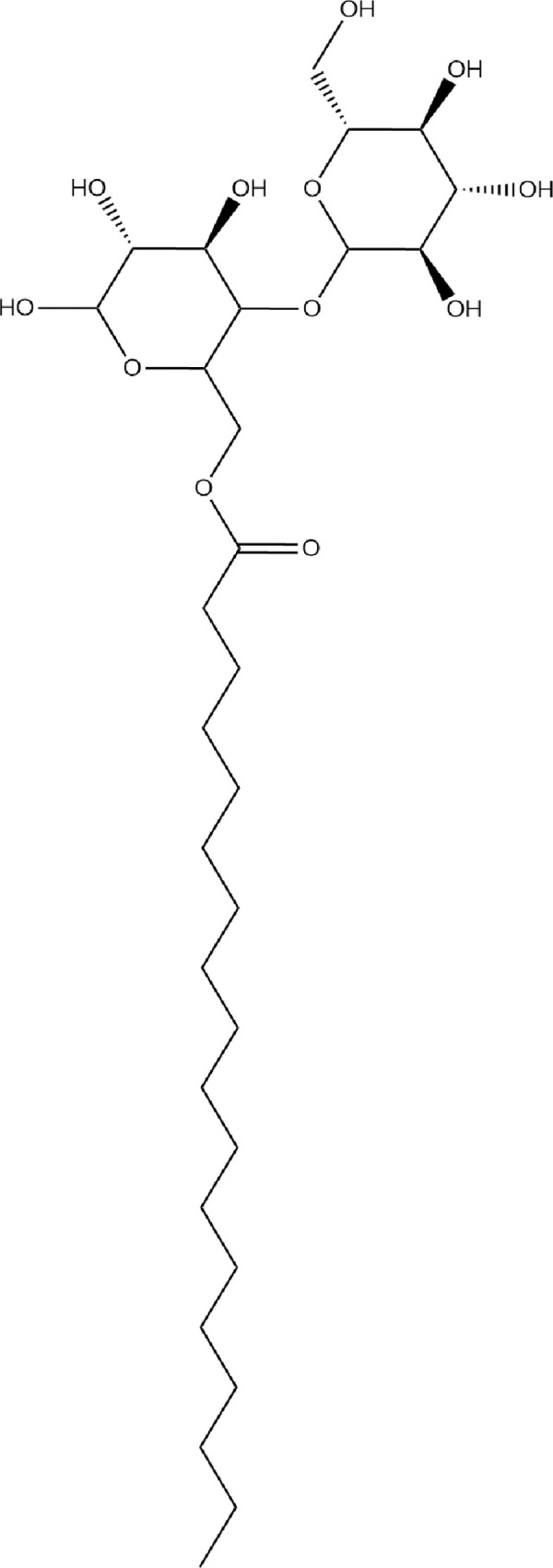
Structure of biosurfactant produced by *Gordonia* sp. IITR100.

All three spectra confirm the extracted surfactant as a glycolipid containing a hydrophilic disaccharide moiety and a hydrophobic octadecanoic acid. The structure of the biosurfactant has been shown in Fig 11.

## Discussion

The biosurfactant production for *Gordonia* sp. IITR100 was confirmed by positive results in drop-collapse assay, oil spreading assay, microplate assay, E_24_ assay and surface tension reduction. Emulsion stability assays at different temperatures, pH values and salt concentrations were performed to determine optimum conditions for biosurfactant action. Higher temperature significantly reduced the stability of the emulsion. It is well known that higher temperature affects the physical properties of the oil (especially viscosity), interfacial films and surfactant properties [46]. The thermal energy of the droplets increases as the temperature rises, as does the frequency of drop collisions. This lowers the interfacial tension, which raises the film drainage rate and promotes faster drop coalescence, lowering emulsion stability [47]. Effect of pH and salt concentration is a result of the change in the concentration of inorganic ions on emulsion stability [48]. The attractive (Van der Walls forces) and repulsive (electrostatic forces) forces that are engaged during particle interaction determine whether colloids coalesce or separate [49]. When salt crystals dissolve in water, they generate their own electrical charges, which adsorb onto the emulsion droplets. The expansion and repulsion of the second layer change as the salt concentration changes, affecting the emulsion’s stability.

*Gordonia* sp. IITR 100 produced a surface-active agent that gave positive results when the TLC plate was stained with iodine and p-anisaldehyde, which confirms the presence of lipids and carbohydrates in the biosurfactant. It gave negative results when stained with ninhydrin which shows the absence of amino acids. LCMS and GCMS studies of the extracted biosurfactant identified it to be a glycolipid with a hydrophilic β-D-glucopyranose, 4-O- β-D-glucopyranosyl group and a hydrophobic octadecanoic acid tail containing an 18-carbon chain. The chemical formula is C_24_H_46_O_7_ and the mass is equal to 630 g/mol. The identity of this compound was confirmed by FTIR, H^1^ NMR and C^13^ NMR of the extracted biosurfactant. Various reports on microbial production of fatty acid esters containing hexoses have indicated they are superior surfactants compared to anionic surfactants such as sodium bis(2-ethylhexyl) sulfosuccinate [1]. While a number of studies report the isolation and application of biosurfactants, there are only limited reports on the chemical identification of biosurfactants produced by microorganisms. This becomes important for field applications where a mixture of different types of biosurfactants would be desirable. The biosurfactant was successful in reducing the surface tension of the media from 61.07 mN/m to 36.82 mN/m. Since the biosurfactant reduced the surface tension of the medium below 45 mN/m, the strain can be considered to be a favourable biosurfactant-producing strain. Biosurfactant production has been reported for other members of *Gordonia*. A glycolipid biosurfactant has been identified from *Gordonia westfalica* [39]. *Gordonia* sp. BS29 has been reported to produce two different surface-active agents; a cell bound glycolipid biosurfactant capable of reducing the surface tension of the medium and another extracellular bioemulsifier capable of producing the stable emulsion but incapable of reducing the surface tension of the medium [50]. Other species of *Gordonia* reported to produce biosurfactants include *Gordonia amicalis* HS-11 [51], *Gordonia nitida* strain LE31 [52], *Gordonia amarae* [9,53,54], *Gordonia alkanivorans* [55], *Gordonia amicalis* LH3 [56], *Gordonia* sp. strain JE-1058 [57], *Gordonia polyisoprenivorans* [58].

The results from emulsion stability tests show that the emulsion is stable at lower temperatures (4°C to 30°C). Lower pH adversely effects the stability of the emulsion, but the emulsion is relatively stable at higher pH up to 10. The emulsion was found to be stable up to a salt concentration of 2%, above which the emulsion index dropped to half. Emulsion stability results indicates that this biosurfactant can be useful in applications like desludging or in oil bioremediation studies. Also, since this strain was isolated from a petroleum-contaminated soil sample, it can be used in a co-culture with other oil-degrading microorganisms to ameliorate the access of the substrate (hydrocarbon mixture) to the microorganisms.

A comparison of the biosurfactant production by *Gordonia* sp. IITR100 with other reported strains of *Gordonia* has been presented in Table 1. All the strains of *Gordonia* have been reported to produce glycolipids. Among the available reports, with the exception of *Gordonia* sp. strain JE-1058, *Gordonia* sp. IITR100 produced the highest concentration of biosurfactant up to 4.01 g/l. The highest concentration of biosurfactant produced was reported by Saeki et al. 2008 and was found out to be equal to 69 g/l [57]. Our studies have reported the second highest concentration of glycolipid biosurfactant. Other strains of *Gordonia* (*Gordonia westfalica* GY40, *Gordonia amicalis*) have been reported to produce as high as 1.85 g/l and 0.53 g/l of biosurfactants [39,58].

**Table 1:** Comparison of biosurfactants produced by various strains of *Gordonia*.

S. No	Microbe	Type of BS	Emulsion stability	Surface tension reduced to	Yield of crude biosurfactant	Reference
1	*Gordonia amarae*	Trehalose lipid	*-*	40 mN/m	68 mg/l	[59]
2	*Gordonia bronchialis*	Lipoglycan	*-*	-	-	[60]
3	*Gordonia* sp. APB	-	-	20.4% reduction	-	[61]
4	*Gordonia alkanivorans*	-	-	33 mN/m	-	[55]
5	*Gordonia rubropertincta*	Lipoglycan	-	-	-	[62]
6	*Gordonia amarae*	Trehalose lipid	-	-	96 mg/l	[53]
7	*Gordonia* sp. M22, BS25, BS29	Glycolipids	-	29.7 mN/m	-	[50]
8	*Gordonia amarae*	-	-	55 mN/m	-	[9]
9	*Gordonia amicalis* LH3	Rhamnolipid	Salt conc. < 5%, Temp = 40°C	38.4 mN/m	-	[56]
10	*Gordonia* sp. strain JE-1058	Glycolipid	-	-	69 g/l	[57]
11	*Gordonia *sp. S14-10	Glycolipid	-	31.6 mN/m	-	[63]
12	*Gordonia *sp. strain BS29	Lipopolysaccharide	-	-	-	[50]
13	*Gordonia* sp. strain JE-1058.	-	-	-	-	[58]
14	*Gordonia amicalis*	Novel class of Lipopolysacchardes	-	37 mN/m and 55 mN/m	0.53 g/l and 0.11 g/l	[64]
15	*Gordonia cholesterolivorans* AMP 10	-	-	24.7 mN/m	-	[65]
16	*Gordonia westfalica *GY40	Glycolipid	Salt conc. 2–10 % Temp 50–121 °C pH value 4–6	35 mN m^−1^	1.85 g/l	[39]
17	*Gordonia amicalis* HS-11	Glycolipid	Temp 30°C pH 7	40 mN/m	0.48 g/l	[51]
18	*Gordonia* sp. 1D (VKM Ac‐2720D)	Trehalose lipid	-	35 mN/m	-	[66]
19	*Gordonia* sp. IITR100	Glycolipid	30°C pH 7	36.83 mN/m	4.01 g/l	This study

The capacity of the biosurfactants produced by different species of *Gordonia* in reducing the surface tension of the culture medium has been widely reported by various research groups. Table 1 also evaluates the performance of various biosurfactants produced by different species of *Gordonia* in terms of their reduction in surface tension of the medium. Biosurfactants from *Gordonia cholesterolivorans* AMP 10 and *Gordonia* sp. BS29 have been reported to reduce the surface tension of the culture medium to 24.7 mN/m and 29.7 mN/m, respectively [50,65]. This is the maximum reduction in surface tension reported for any of the biosurfactants produced by *Gordonia*. Most of the biosurfactants from *Gordonia* have been reported to reduce the surface tension of the culture medium to between 30 mN/m and 40 mN/m [39,50,51,55,56,58,63]. In our study, we found *Gordonia* sp. IITR100 to be successful in reducing the surface tension of the culture medium from 61.06 mN/m to 36.82 mN/m.

Bioinformatic analysis of the genome sequence of *Gordonia* sp. IITR100 confirmed the presence of all the genes required for biosynthesis of the glycolipids. Similar to rhamnolipid biosynthesis, glycolipid biosynthesis in *Gordonia* sp. IITR100 appears to occur in three steps: synthesis of a long fatty acid backbone from acyl-CoA, formation of a carbohydrate moiety from D-glucose and coupling of the carbohydrate moiety with the fatty acid backbone to form the glycolipid biosurfactant. KEGG pathway analysis showed that biosynthesis of C18 fatty acid chains from acetyl Co-A in *Gordonia terrae* and *Gordonia alkanivorans* is carried out by various enzymes which include acetyl-CoA ligase, ACP S-malonyl-transferase, acetyl-CoA-acetyltransferase, acyltransferase, NADP+ oxidoreductase, beta-hydroxyacyl-ACP dehydratase and NAD+ oxidoreductase. The enzymes involved in conversion of dTDP D-glucose to form the carbohydrate moiety of the biosurfactant (e.g., dTDP L-rhamnose) include glucose-1- phosphate thymidylyltransferase, dTDPD-glucose 4,6-dehydratase, dTDP-6-deoxyD-xylo-4-hexulose 3,5-epimerase and dTDP-6-deoxy-L-lyxo-4-hexulose reductase [67]. The last steps in glycolipid biosynthesis- transfer of the carbohydrate moiety to the fatty acid backbone- is carried out by various transferases like rhamnosyltransferases and glycosyltransferases. Analysis of the genome sequence of *Gordonia* sp. IITR100 revealed the presence of genes for all the important enzymes required for glycolipid biosynthesis. The details of the genes and their gene numbers are given in Tables 2–4.

**Table 2:** Genes involved in biosynthesis of C18 fatty acid chain.

S No.	Sequence name	Sequence description
1	Gene_311	fatty-acid—ligase
2	Gene_617	long-chain-fatty-acid—ligase
3	Gene_1405	long-chain-fatty-acid—ligase
4	Gene_1849	long-chain-fatty-acid—ligase
5	Gene_2122	acyl—ligase
6	Gene_2401	long-chain-fatty-acid—ligase
7	Gene_2869	long-chain-fatty-acid—ligase
8	Gene_2959	long-chain-fatty-acid—ligase
9	Gene_3734	fatty acid—ligase
10	Gene_3780	fatty-acid—ligase
11	Gene_4000	fatty-acid—ligase
12	Gene_4419	fatty-acid—ligase
13	Gene_71	MULTISPECIES: long-chain-fatty-acid—ligase
14	Gene_2952	ACP S-malonyltransferase
15	Gene_465	acetyl- acetyltransferase
16	Gene_1403	acetyl- acetyltransferase
17	Gene_1408	acetyl- acetyltransferase
18	Gene_1515	acetyl- acetyltransferase
19	Gene_1815	acetyl- acetyltransferase
20	Gene_2508	acetyl- acetyltransferase
21	Gene_3181	acetyl- acetyltransferase
22	Gene_3925	acetyl- acetyltransferase
23	Gene_4178	acetyl- acetyltransferase
24	Gene_4234	acetyl- acetyltransferase
25	Gene_4414	acetyl- acetyltransferase
26	Gene_212	acyltransferase
27	Gene_291	acyltransferase
28	Gene_292	acyltransferase
29	Gene_1256	acyltransferase
30	Gene_1963	acyltransferase
31	Gene_2346	acyltransferase
32	Gene_2351	acyltransferase
33	Gene_2436	acyltransferase
34	Gene_2644	acyltransferase
35	Gene_2798	acyltransferase
36	Gene_3383	acyltransferase
37	Gene_3599	acyltransferase
38	Gene_4067	acyltransferase
39	Gene_1724	NAD(P)-dependent oxidoreductase
40	Gene_2967	NAD(P)-dependent oxidoreductase
41	Gene_3944	NAD(P)-dependent oxidoreductase
42	Gene_4108	NADP oxidoreductase
43	Gene_4423	NADP-dependent oxidoreductase
44	Gene_53	oxidoreductase
45	Gene_435	oxidoreductase
46	Gene_519	oxidoreductase
47	Gene_702	oxidoreductase
48	Gene_810	oxidoreductase
49	Gene_1227	oxidoreductase
50	Gene_1241	oxidoreductase
51	Gene_2121	oxidoreductase
52	Gene_2215	oxidoreductase
53	Gene_2310	oxidoreductase
54	Gene_2407	oxidoreductase
55	Gene_2420	oxidoreductase
56	Gene_2588	oxidoreductase
57	Gene_2712	oxidoreductase
58	Gene_3186	oxidoreductase
59	Gene_3213	oxidoreductase
60	Gene_3487	oxidoreductase
61	Gene_3738	oxidoreductase
62	Gene_3745	oxidoreductase
63	Gene_3920	oxidoreductase
64	Gene_4003	oxidoreductase
65	Gene_4029	oxidoreductase
66	Gene_4384	oxidoreductase
67	Gene_4405	oxidoreductase
68	Gene_4531	oxidoreductase
69	Gene_4578	oxidoreductase
70	Gene_3936	acyl dehydratase
71	Gene_818	beta-hydroxyacyl-ACP dehydratase

**Table 3:** Genes involved in biosynthesis of carbohydrate moiety.

S No.	Sequence name	Sequence description
1	Gene_2014	polyribonucleotide nucleotidyltransferase
2	Gene_581	glucose-1-phosphate thymidylyltransferase
3	Gene_1077	glucose-1-phosphate thymidylyltransferase
4	Gene_1078	dTDP-glucose 4,6-dehydratase
5	Gene_580	dTDP-glucose 4,6-dehydratase
6	Gene_2199	ribulose-phosphate 3-epimerase
7	Gene_2427	nucleoside-diphosphate sugar epimerase
8	Gene_3845	ribulose phosphate epimerase
9	Gene_579	UDP-glucose 4-epimerase
10	Gene_582	dTDP-4-dehydrorhamnose 3,5-epimerase
11	Gene_236	Glucose dehydrogenase sorbosone dehydrogenase
12	Gene_1081	UDP-glucose 6-dehydrogenase

**Table 4:** Genes involved in linking lipid chain to carbohydrate moiety to form glycolipid.

S No.	Sequence name	Sequence description
1	Gene_1725	N-acetylglucosaminyl-diphospho-decaprenol L-rhamnosyltransferase
2	Gene_1764	glycosyl transferase
3	Gene_2044	glycosyl transferase
4	Gene_2127	glycosyl transferase
5	Gene_2338	mannosyltransferase
6	Gene_2345	glycosyl transferase family 1
7	Gene_2704	glycosyl transferase
8	Gene_2862	mannosyltransferase
9	Gene_3450	glycosyl transferase
10	Gene_3452	glycosyl transferase
11	Gene_3455	glycosyl transferase family 1
12	Gene_3499	glycosyl transferase
13	Gene_3500	phosphoribosyltransferase
14	Gene_3618	glycosyl transferase family 1
15	Gene_3948	glycosyl transferase family 1
16	Gene_4260	glycosyl transferase
17	Gene_4264	glycosyl transferase
18	Gene_4497	glycosyl transferase
19	Gene_300	glycosyl transferase
20	Gene_302	glycosyl transferase
21	Gene_360	galactofuranosyl transferase
22	Gene_445	glycosyl transferase
23	Gene_588	glycosyl transferase
24	Gene_589	glycosyl transferase family 2
25	Gene_771	glycosyl transferase
26	Gene_1084	glycosyl transferase
27	Gene_1085	glycosyl transferase
28	Gene_1087	glycosyl transferase
29	Gene_1099	glycosyl transferase
30	Gene_1100	glycosyl transferase
31	Gene_1759	glycosyl transferase
32	Gene_1760	glycosyl transferase
33	Gene_1761	glycosyl transferase

Based on the yield and the surface tension reduction capacity, the biosurfactant produced by *Gordonia* sp. IITR100 can be useful in a wide range of applications like oil solubilization, desludging of oil containers and enhancement of oil biodesulfurization and biodegradation.
